# *cis*,*cis*,*cis*-(Acetato-κ^2^ *O*,*O*′)bis­[1,2-bis­(diphenyl­phosphan­yl)ethane-κ^2^ *P*,*P*′]ruthenium(II) 0.75-trifluoro­methane­sulfonate 0.25-chloride

**DOI:** 10.1107/S160053681300737X

**Published:** 2013-03-23

**Authors:** João Figueira, João Rodrigues, Arto Valkonen

**Affiliations:** a CQM – Centro de Química da Madeira, MMRG, Universidade da Madeira, Campus Universitário da Penteada, 9000-390 Funchal, Portugal; b University of Jyväskylä, Department of Chemistry, PO Box 35, FIN-40014 Jyväskylä, Finland

## Abstract

In the title Ru^II^ carboxyl­ate compound, [Ru(C_2_H_3_O_2_)(C_26_H_24_P_2_)_2_](CF_3_O_3_S)_0.75_Cl_0.25_, the distorted tris-bidentate octa­hedral stereochemistry about the Ru^II^ atom in the complex cation comprises four P-atom donors from two 1,2-bis­(diphenyl­phosphan­yl)ethane ligands [Ru—P = 2.2881 (13)–2.3791 (13) Å] and two O-atom donors from the acetate ligand [Ru—O = 2.191 (3) and 2.202 (3) Å]. The disordered counter-anions are located on the same site in the structure in a 3:1 ratio, the expanded formula comprising four complex cations, three trifluoro­methane­sulfonate anions and one chloride anion, with two such formula units in the unit cell.

## Related literature

For applications of Ru^II^ carboxyl­ate complexes, see: Kilbas *et al.* (2012 ▶); Mikuriya *et al.* (2011 ▶); Hiett *et al.* (2011 ▶); Liu *et al.* (2012 ▶). For similar complexes, see: Holle *et al.* (1997 ▶); Wyman *et al.* (2004 ▶); Lucas *et al.* (2000 ▶).
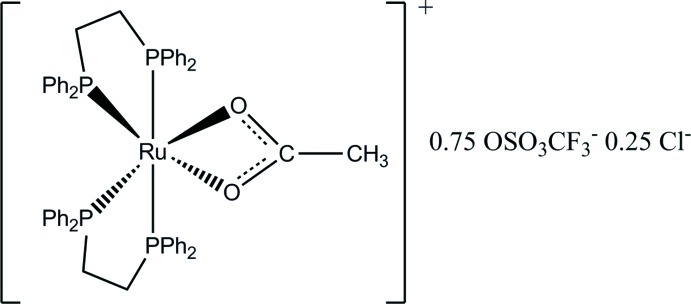


## Experimental

### 

#### Crystal data

[Ru(C_2_H_3_O_2_)(C_26_H_24_P_2_)_2_](CF_3_O_3_S)_0.75_Cl_0.25_
*M* *_r_* = 4310.25
Orthorhombic, 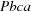

*a* = 15.7463 (2) Å
*b* = 21.8914 (3) Å
*c* = 28.6122 (4) Å
*V* = 9862.9 (2) Å^3^
*Z* = 2
Mo *K*α radiation
μ = 0.55 mm^−1^
*T* = 173 K
0.25 × 0.20 × 0.15 mm

#### Data collection

Bruker–Nonius KappaCCD diffractometer with APEXII detector
Absorption correction: multi-scan (*DENZO-SMN*; Otwinowski & Minor, 1997 ▶) *T*_min_ = 0.875, *T*_max_ = 0.922
16563 measured reflections
8662 independent reflections
7095 reflections with *I* > 2σ(*I*)
*R*_int_ = 0.036

#### Refinement

*R*[*F* ^2^ > 2σ(*F* ^2^)] = 0.061
*wR*(*F* ^2^) = 0.148
*S* = 1.13
8662 reflections
626 parameters
7 restraints
H-atom parameters constrained
Δρ_max_ = 0.90 e Å^−3^
Δρ_min_ = −1.04 e Å^−3^

### 

Data collection: *COLLECT* (Bruker, 2004 ▶); cell refinement: *DENZO-SMN* (Otwinowski & Minor, 1997 ▶); data reduction: *DENZO-SMN*; program(s) used to solve structure: *SHELXS86* (Sheldrick, 2008 ▶); program(s) used to refine structure: *SHELXL97* (Sheldrick, 2008 ▶); molecular graphics: *ORTEP-3 for Windows* (Farrugia, 2012 ▶); software used to prepare material for publication: *WinGX* (Farrugia, 2012 ▶).

## Supplementary Material

Crystal structure: contains datablock(s) global, I. DOI: 10.1107/S160053681300737X/zs2249sup1.cif

Structure factors: contains datablock(s) I. DOI: 10.1107/S160053681300737X/zs2249Isup2.hkl

Additional supplementary materials:  crystallographic information; 3D view; checkCIF report

## Figures and Tables

**Fig. 1.**
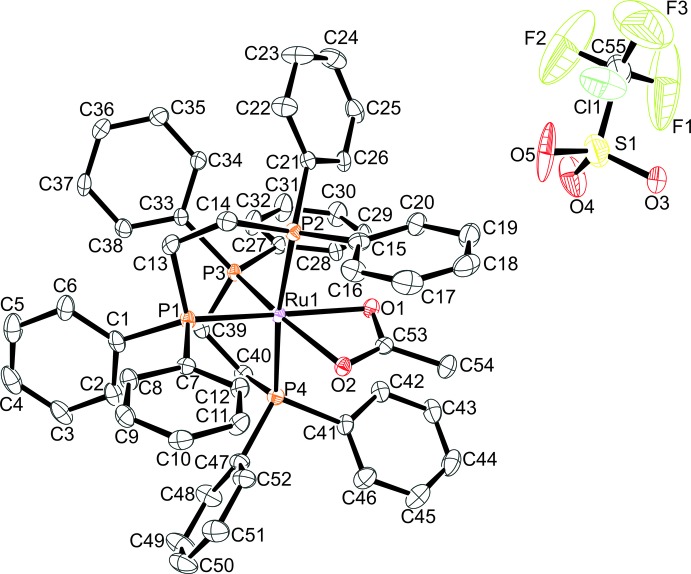
Plot of the title compound, with atom labels and 30% probability displacement ellipsoids for non-H atoms.

## supplementary crystallographic information

### Comment

Ruthenium carboxylate complexes have found increasing interest as building blocks for larger macrocycles (Kilbas *et al.*, 2012), preparation of magnetic materials (Mikuriya *et al.*, 2011), as catalysts (Hiett *et al.*, 2011) and as promising materials for NLO devices (Liu *et al.*, 2012).

In the Ru^II^ carboxylate compound, *cis*-[Ru(η^2^(CH_3_CO_2_) (C_26_H_24_P_2_)_2_] (CF_3_O_3_S)_0.75_ Cl_0.25_, the title compound (Fig. 1) the complex cation is formed by three bidentate chelate ligands (two 1,2-bis(diphenylphosphanyl)ethane ligands and one acetato ligand) giving bite angles at the metal of 82.36 (5) and 83.44 (5)° for the phosphines and 59.18 (12)° for the acetate. The observed Ru—P and Ru—O bond distances [range 2.2882 (13)–2.3791 (13) Å and 2.191 (3), 2.202 (3) Å, respectively] result in a distorted octahedral geometry consistent with similar complexes having the *cis*-configuration (Holle *et al.*, 1997, Lucas *et al.*, 2000, Wyman *et al.*, 2004). The Ru—P bond distances in the title complex are shorter than those in complexes in which the acetate group is *trans*-related but are well within reported ranges (Holle *et al.*, 1997, Lucas *et al.*, 2000, Wyman *et al.*, 2004).

The disordered counter anions are located on the same site in the structure in a 3:1 ratio, the complete complex unit having the formula 4[Ru(C_2_H_3_O_2_)(C_26_H_24_P_2_)_2_] (CF_3_O_3_S)_3_ Cl, with two such formula units in the unit cell.

### Experimental

The title compound was obtained as a by-product in the attempted crystallization of the products of the reaction of [RuCl(dppe)_2_][PF_6_] and 1,4-diheptoxy-2,5-diethynylbenzene, using a dichloromethane–diethyl ether solvent mixture at -20 °C.

### Refinement

All H atoms were visible in electron density maps, but they were included at calculated positions and allowed to ride on the C atoms with C—H = 0.95 Å (aromatic), 0.98 Å (methyl) and 0.99 Å (methylene), with *U*_iso_(H) = 1.2 (or 1.5 for methyl) times *U*_eq_(C). Two positions for each of the methyl protons on C54 were found and this disorder was handled by refining these positions constrained over 60° rotational sites with 0.5 site occupancy. The trifluoromethanesulfonate and chloride anions were found to occupy a common site, with occupancies of 0.75 and 0.25, respectively. Small geometrical disorder in the trifluoromethanesulfonate anion was handled by restraining the C—F (1.320 Å), S—C (1.820 Å) and F···F (2.150 Å) distances (s = 0.001). The anisotropic displacement parameters were made equal for S1 and C55.

### Crystal data

**Table:**

[Ru(C_2_H_3_O_2_)(C_26_H_24_P_2_)_2_](CF_3_O_3_S)_0.75_Cl_0.25_	*F*(000) = 4432
*M**_r_* = 4310.25	*D*_x_ = 1.451 Mg m^−^^3^
Orthorhombic, *P**b**c**a*	Mo *K*α radiation, λ = 0.71073 Å
Hall symbol: -P 2ac 2ab	Cell parameters from 137403 reflections
*a* = 15.7463 (2) Å	θ = 2.9–28.3°
*b* = 21.8914 (3) Å	µ = 0.55 mm^−^^1^
*c* = 28.6122 (4) Å	*T* = 173 K
*V* = 9862.9 (2) Å^3^	Prism, green-yellow
*Z* = 2	0.25 × 0.20 × 0.15 mm

### Data collection

**Table:**

Bruker–Nonius KappaCCD diffractometer with APEXII detector	8662 independent reflections
Radiation source: fine-focus sealed tube	7095 reflections with *I* > 2σ(*I*)
Graphite monochromator	*R*_int_ = 0.036
Detector resolution: 9 pixels mm^-1^	θ_max_ = 25.0°, θ_min_ = 3.0°
CCD rotation images, thick slices scans	*h* = −18→18
Absorption correction: multi-scan (*DENZO-SMN*; Otwinowski & Minor, 1997)	*k* = −25→26
*T*_min_ = 0.875, *T*_max_ = 0.922	*l* = −33→34
16563 measured reflections	

### Refinement

**Table:**

Refinement on *F*^2^	Primary atom site location: structure-invariant direct methods
Least-squares matrix: full	Secondary atom site location: difference Fourier map
*R*[*F*^2^ > 2σ(*F*^2^)] = 0.061	Hydrogen site location: inferred from neighbouring sites
*wR*(*F*^2^) = 0.148	H-atom parameters constrained
*S* = 1.13	*w* = 1/[σ^2^(*F*_o_^2^) + (0.0484*P*)^2^ + 43.975*P*] where *P* = (*F*_o_^2^ + 2*F*_c_^2^)/3
8662 reflections	(Δ/σ)_max_ = 0.001
626 parameters	Δρ_max_ = 0.90 e Å^−^^3^
7 restraints	Δρ_min_ = −1.04 e Å^−^^3^

### Special details

**Table:**

Geometry. All s.u.'s (except the s.u. in the dihedral angle between two l.s. planes) are estimated using the full covariance matrix. The cell s.u.'s are taken into account individually in the estimation of s.u.'s in distances, angles and torsion angles; correlations between s.u.'s in cell parameters are only used when they are defined by crystal symmetry. An approximate (isotropic) treatment of cell s.u.'s is used for estimating s.u.'s involving l.s. planes.
Refinement. Refinement of *F*^2^ against ALL reflections. The weighted *R*-factor *wR* and goodness of fit *S* are based on *F*^2^, conventional *R*-factors *R* are based on *F*, with *F* set to zero for negative *F*^2^. The threshold expression of *F*^2^ > 2σ(*F*^2^) is used only for calculating *R*-factors(gt) *etc*. and is not relevant to the choice of reflections for refinement. *R*-factors based on *F*^2^ are statistically about twice as large as those based on *F*, and *R*- factors based on ALL data will be even larger.

### Fractional atomic coordinates and isotropic or equivalent isotropic displacement parameters (Å^2^)

**Table:**

	*x*	*y*	*z*	*U*_iso_*/*U*_eq_	Occ. (<1)
Ru1	0.24211 (2)	0.986017 (17)	0.615267 (13)	0.02161 (12)	
P1	0.21047 (8)	1.02120 (6)	0.68978 (4)	0.0250 (3)	
P2	0.38185 (8)	0.98385 (6)	0.64581 (4)	0.0256 (3)	
P3	0.23401 (8)	1.08350 (6)	0.58676 (4)	0.0234 (3)	
P4	0.10573 (8)	0.97601 (6)	0.58030 (5)	0.0268 (3)	
O1	0.2933 (2)	0.93092 (15)	0.55788 (12)	0.0289 (8)	
O2	0.2383 (2)	0.88552 (15)	0.61809 (11)	0.0268 (8)	
C1	0.1295 (3)	1.0776 (2)	0.70689 (18)	0.0318 (12)	
C2	0.0460 (4)	1.0689 (3)	0.6930 (2)	0.0388 (13)	
H2	0.0323	1.0349	0.6738	0.047*	
C3	−0.0182 (4)	1.1088 (3)	0.7068 (2)	0.0468 (16)	
H3	−0.0747	1.1028	0.6962	0.056*	
C4	0.0006 (5)	1.1572 (3)	0.7358 (2)	0.0533 (18)	
H4	−0.0429	1.1842	0.7458	0.064*	
C5	0.0833 (5)	1.1659 (3)	0.7503 (2)	0.0517 (17)	
H5	0.0963	1.1989	0.7706	0.062*	
C6	0.1477 (4)	1.1270 (2)	0.73574 (19)	0.0400 (14)	
H6	0.2045	1.1342	0.7455	0.048*	
C7	0.1886 (3)	0.9611 (2)	0.73337 (16)	0.0255 (11)	
C8	0.1589 (3)	0.9772 (2)	0.77776 (17)	0.0314 (12)	
H8	0.1493	1.0189	0.7850	0.038*	
C9	0.1434 (3)	0.9330 (3)	0.81120 (18)	0.0339 (12)	
H9	0.1229	0.9445	0.8411	0.041*	
C10	0.1576 (3)	0.8718 (3)	0.80126 (18)	0.0351 (13)	
H10	0.1457	0.8412	0.8239	0.042*	
C11	0.1893 (4)	0.8560 (2)	0.75789 (18)	0.0346 (13)	
H11	0.2015	0.8144	0.7512	0.042*	
C12	0.2035 (3)	0.8997 (2)	0.72447 (17)	0.0295 (11)	
H12	0.2240	0.8877	0.6946	0.035*	
C13	0.3081 (3)	1.0540 (2)	0.71472 (17)	0.0297 (12)	
H13A	0.3216	1.0932	0.6992	0.036*	
H13B	0.3004	1.0618	0.7485	0.036*	
C14	0.3806 (3)	1.0085 (3)	0.70718 (17)	0.0329 (12)	
H14A	0.3727	0.9726	0.7278	0.039*	
H14B	0.4355	1.0279	0.7152	0.039*	
C15	0.4262 (3)	0.9063 (2)	0.64697 (19)	0.0315 (12)	
C16	0.4122 (4)	0.8679 (3)	0.6845 (2)	0.0433 (14)	
H16	0.3824	0.8826	0.7111	0.052*	
C17	0.4413 (4)	0.8082 (3)	0.6836 (3)	0.0560 (18)	
H17	0.4316	0.7825	0.7098	0.067*	
C18	0.4840 (4)	0.7855 (3)	0.6453 (3)	0.0528 (18)	
H18	0.5028	0.7442	0.6448	0.063*	
C19	0.4992 (4)	0.8237 (3)	0.6078 (3)	0.0502 (16)	
H19	0.5291	0.8087	0.5813	0.060*	
C20	0.4711 (4)	0.8837 (2)	0.6086 (2)	0.0375 (13)	
H20	0.4825	0.9097	0.5827	0.045*	
C21	0.4649 (3)	1.0279 (2)	0.61657 (17)	0.0265 (11)	
C22	0.5263 (4)	1.0605 (3)	0.6399 (2)	0.0424 (14)	
H22	0.5266	1.0610	0.6731	0.051*	
C23	0.5880 (4)	1.0928 (3)	0.6155 (3)	0.0597 (19)	
H23	0.6311	1.1142	0.6321	0.072*	
C24	0.5868 (4)	1.0939 (3)	0.5674 (3)	0.0537 (17)	
H24	0.6285	1.1165	0.5507	0.064*	
C25	0.5254 (4)	1.0622 (3)	0.5436 (2)	0.0428 (15)	
H25	0.5245	1.0630	0.5104	0.051*	
C26	0.4646 (3)	1.0292 (2)	0.56764 (18)	0.0316 (12)	
H26	0.4224	1.0072	0.5509	0.038*	
C27	0.2689 (3)	1.0974 (2)	0.52628 (17)	0.0283 (11)	
C28	0.2936 (3)	1.0509 (2)	0.49636 (17)	0.0287 (11)	
H28	0.2979	1.0102	0.5077	0.034*	
C29	0.3120 (4)	1.0635 (3)	0.44953 (19)	0.0384 (13)	
H29	0.3296	1.0315	0.4293	0.046*	
C30	0.3045 (5)	1.1219 (3)	0.4330 (2)	0.0507 (17)	
H30	0.3157	1.1301	0.4010	0.061*	
C31	0.2809 (5)	1.1685 (3)	0.4622 (2)	0.0565 (19)	
H31	0.2770	1.2091	0.4505	0.068*	
C32	0.2627 (4)	1.1568 (3)	0.5087 (2)	0.0448 (15)	
H32	0.2460	1.1892	0.5288	0.054*	
C33	0.2783 (3)	1.1506 (2)	0.61595 (16)	0.0230 (10)	
C34	0.3646 (3)	1.1632 (2)	0.6107 (2)	0.0330 (12)	
H34	0.3982	1.1381	0.5910	0.040*	
C35	0.4020 (4)	1.2121 (2)	0.6338 (2)	0.0385 (13)	
H35	0.4610	1.2199	0.6304	0.046*	
C36	0.3526 (4)	1.2493 (2)	0.6619 (2)	0.0382 (13)	
H36	0.3776	1.2828	0.6779	0.046*	
C37	0.2670 (4)	1.2376 (2)	0.6667 (2)	0.0376 (13)	
H37	0.2333	1.2634	0.6860	0.045*	
C38	0.2296 (3)	1.1890 (2)	0.64379 (17)	0.0313 (12)	
H38	0.1704	1.1818	0.6471	0.038*	
C39	0.1203 (3)	1.1014 (2)	0.57906 (18)	0.0296 (11)	
H39A	0.0926	1.1061	0.6099	0.036*	
H39B	0.1138	1.1401	0.5616	0.036*	
C40	0.0794 (3)	1.0492 (2)	0.55215 (18)	0.0316 (12)	
H40A	0.1003	1.0492	0.5195	0.038*	
H40B	0.0170	1.0547	0.5515	0.038*	
C41	0.0981 (3)	0.9187 (2)	0.53316 (18)	0.0321 (12)	
C42	0.1437 (3)	0.9273 (3)	0.49147 (19)	0.0354 (13)	
H42	0.1761	0.9635	0.4871	0.042*	
C43	0.1415 (4)	0.8832 (3)	0.4565 (2)	0.0426 (15)	
H43	0.1719	0.8896	0.4282	0.051*	
C44	0.0952 (4)	0.8303 (3)	0.4626 (2)	0.0533 (18)	
H44	0.0940	0.8001	0.4387	0.064*	
C45	0.0508 (5)	0.8214 (3)	0.5037 (3)	0.0588 (19)	
H45	0.0189	0.7850	0.5078	0.071*	
C46	0.0521 (4)	0.8646 (3)	0.5387 (2)	0.0468 (15)	
H46	0.0215	0.8576	0.5668	0.056*	
C47	0.0162 (3)	0.9537 (3)	0.61700 (18)	0.0330 (12)	
C48	−0.0656 (4)	0.9769 (3)	0.6110 (2)	0.0489 (16)	
H48	−0.0772	1.0049	0.5865	0.059*	
C49	−0.1296 (4)	0.9587 (4)	0.6410 (2)	0.065 (2)	
H49	−0.1850	0.9752	0.6374	0.077*	
C50	−0.1147 (4)	0.9174 (4)	0.6759 (2)	0.0580 (19)	
H50	−0.1595	0.9056	0.6962	0.070*	
C51	−0.0346 (4)	0.8929 (3)	0.6816 (2)	0.0502 (16)	
H51	−0.0243	0.8637	0.7055	0.060*	
C52	0.0317 (4)	0.9112 (3)	0.6520 (2)	0.0400 (14)	
H52	0.0870	0.8945	0.6559	0.048*	
C53	0.2719 (3)	0.8818 (2)	0.57815 (17)	0.0269 (11)	
C54	0.2851 (4)	0.8208 (2)	0.5550 (2)	0.0386 (13)	
H54A	0.3287	0.8246	0.5307	0.058*	0.50
H54B	0.2317	0.8073	0.5408	0.058*	0.50
H54C	0.3035	0.7909	0.5783	0.058*	0.50
H54D	0.2472	0.7906	0.5692	0.058*	0.50
H54E	0.3442	0.8079	0.5591	0.058*	0.50
H54F	0.2724	0.8243	0.5215	0.058*	0.50
S1	0.71959 (16)	0.70092 (13)	0.66161 (10)	0.0689 (7)	0.75
F1	0.8095 (8)	0.7154 (5)	0.5899 (3)	0.299 (12)	0.75
F2	0.8041 (9)	0.7953 (3)	0.6335 (4)	0.279 (10)	0.75
F3	0.8855 (5)	0.7204 (6)	0.6522 (3)	0.225 (6)	0.75
O3	0.7261 (3)	0.6357 (2)	0.6583 (2)	0.0504 (15)	0.75
O4	0.6611 (4)	0.7297 (4)	0.6355 (2)	0.081 (2)	0.75
O5	0.7051 (7)	0.7148 (3)	0.7085 (2)	0.103 (3)	0.75
C55	0.8117 (3)	0.7352 (3)	0.6335 (2)	0.0689 (7)	0.75
Cl1	0.8005 (6)	0.7210 (6)	0.6672 (4)	0.108 (4)	0.25

### Atomic displacement parameters (Å^2^)

**Table:**

	*U*^11^	*U*^22^	*U*^33^	*U*^12^	*U*^13^	*U*^23^
Ru1	0.0256 (2)	0.0248 (2)	0.0145 (2)	0.00014 (16)	−0.00011 (15)	−0.00065 (16)
P1	0.0301 (7)	0.0292 (7)	0.0157 (6)	0.0006 (5)	0.0028 (5)	−0.0011 (5)
P2	0.0269 (6)	0.0310 (7)	0.0188 (6)	0.0010 (6)	−0.0008 (5)	−0.0006 (5)
P3	0.0282 (7)	0.0255 (6)	0.0164 (6)	0.0016 (5)	0.0026 (5)	−0.0005 (5)
P4	0.0275 (7)	0.0320 (7)	0.0210 (7)	−0.0015 (5)	−0.0032 (5)	−0.0002 (6)
O1	0.0340 (19)	0.0292 (19)	0.0236 (19)	0.0007 (15)	−0.0014 (15)	−0.0037 (15)
O2	0.0330 (19)	0.0274 (18)	0.0202 (18)	0.0001 (15)	−0.0003 (15)	−0.0016 (14)
C1	0.042 (3)	0.032 (3)	0.021 (3)	0.004 (2)	0.013 (2)	0.007 (2)
C2	0.045 (3)	0.042 (3)	0.030 (3)	0.007 (3)	0.010 (3)	0.001 (3)
C3	0.044 (3)	0.052 (4)	0.045 (4)	0.011 (3)	0.019 (3)	0.014 (3)
C4	0.068 (5)	0.044 (4)	0.048 (4)	0.018 (3)	0.030 (4)	0.016 (3)
C5	0.081 (5)	0.032 (3)	0.042 (4)	0.015 (3)	0.012 (3)	0.001 (3)
C6	0.066 (4)	0.029 (3)	0.025 (3)	0.002 (3)	0.011 (3)	0.002 (2)
C7	0.025 (2)	0.033 (3)	0.019 (2)	−0.004 (2)	−0.003 (2)	0.002 (2)
C8	0.036 (3)	0.036 (3)	0.021 (3)	0.002 (2)	0.004 (2)	0.001 (2)
C9	0.037 (3)	0.044 (3)	0.021 (3)	0.002 (2)	0.005 (2)	0.002 (2)
C10	0.038 (3)	0.045 (3)	0.022 (3)	−0.013 (3)	−0.003 (2)	0.007 (2)
C11	0.050 (3)	0.032 (3)	0.021 (3)	−0.007 (3)	−0.004 (2)	0.001 (2)
C12	0.038 (3)	0.038 (3)	0.012 (2)	−0.003 (2)	0.001 (2)	−0.002 (2)
C13	0.041 (3)	0.034 (3)	0.014 (2)	−0.006 (2)	0.000 (2)	−0.003 (2)
C14	0.033 (3)	0.047 (3)	0.019 (3)	−0.006 (2)	−0.003 (2)	−0.001 (2)
C15	0.027 (3)	0.032 (3)	0.035 (3)	−0.002 (2)	−0.006 (2)	0.005 (2)
C16	0.038 (3)	0.044 (3)	0.048 (4)	0.001 (3)	0.001 (3)	0.010 (3)
C17	0.048 (4)	0.045 (4)	0.075 (5)	−0.001 (3)	−0.005 (4)	0.025 (4)
C18	0.045 (4)	0.026 (3)	0.087 (5)	0.001 (3)	−0.017 (4)	0.005 (3)
C19	0.048 (4)	0.039 (3)	0.063 (4)	0.006 (3)	−0.005 (3)	−0.012 (3)
C20	0.046 (3)	0.030 (3)	0.037 (3)	0.003 (3)	−0.002 (3)	0.002 (2)
C21	0.026 (3)	0.027 (3)	0.026 (3)	0.005 (2)	0.001 (2)	0.000 (2)
C22	0.037 (3)	0.056 (4)	0.034 (3)	−0.007 (3)	−0.006 (3)	0.010 (3)
C23	0.041 (4)	0.080 (5)	0.058 (5)	−0.025 (3)	−0.008 (3)	0.017 (4)
C24	0.042 (4)	0.057 (4)	0.062 (5)	−0.009 (3)	0.015 (3)	0.019 (4)
C25	0.048 (4)	0.040 (3)	0.040 (4)	0.008 (3)	0.019 (3)	0.006 (3)
C26	0.038 (3)	0.028 (3)	0.029 (3)	0.005 (2)	0.007 (2)	−0.004 (2)
C27	0.034 (3)	0.030 (3)	0.021 (3)	−0.001 (2)	0.001 (2)	−0.001 (2)
C28	0.031 (3)	0.031 (3)	0.024 (3)	0.006 (2)	0.005 (2)	−0.003 (2)
C29	0.049 (3)	0.042 (3)	0.024 (3)	0.005 (3)	0.011 (3)	−0.005 (2)
C30	0.080 (5)	0.051 (4)	0.021 (3)	−0.004 (3)	0.015 (3)	0.006 (3)
C31	0.102 (6)	0.037 (3)	0.030 (3)	0.001 (4)	0.021 (3)	0.005 (3)
C32	0.077 (4)	0.031 (3)	0.027 (3)	0.002 (3)	0.011 (3)	0.002 (2)
C33	0.027 (2)	0.023 (2)	0.019 (2)	0.003 (2)	0.001 (2)	0.001 (2)
C34	0.035 (3)	0.025 (3)	0.039 (3)	0.000 (2)	0.006 (2)	−0.005 (2)
C35	0.035 (3)	0.032 (3)	0.048 (4)	−0.003 (2)	0.001 (3)	−0.004 (3)
C36	0.047 (3)	0.029 (3)	0.039 (3)	−0.004 (3)	−0.003 (3)	−0.008 (2)
C37	0.049 (4)	0.032 (3)	0.032 (3)	0.005 (3)	0.006 (3)	−0.012 (2)
C38	0.037 (3)	0.034 (3)	0.023 (3)	−0.001 (2)	0.008 (2)	0.002 (2)
C39	0.031 (3)	0.032 (3)	0.026 (3)	0.005 (2)	0.002 (2)	0.001 (2)
C40	0.030 (3)	0.041 (3)	0.024 (3)	0.001 (2)	−0.002 (2)	0.002 (2)
C41	0.032 (3)	0.038 (3)	0.027 (3)	0.006 (2)	−0.009 (2)	−0.007 (2)
C42	0.035 (3)	0.044 (3)	0.027 (3)	0.007 (2)	−0.007 (2)	−0.003 (2)
C43	0.040 (3)	0.060 (4)	0.028 (3)	0.014 (3)	−0.010 (2)	−0.011 (3)
C44	0.060 (4)	0.048 (4)	0.052 (4)	0.010 (3)	−0.022 (3)	−0.020 (3)
C45	0.071 (5)	0.043 (4)	0.062 (5)	−0.010 (3)	−0.011 (4)	−0.009 (3)
C46	0.059 (4)	0.042 (3)	0.039 (4)	−0.010 (3)	−0.003 (3)	−0.001 (3)
C47	0.028 (3)	0.044 (3)	0.028 (3)	−0.008 (2)	−0.001 (2)	−0.002 (2)
C48	0.037 (3)	0.076 (5)	0.034 (3)	−0.002 (3)	−0.004 (3)	0.011 (3)
C49	0.031 (3)	0.118 (6)	0.044 (4)	−0.004 (4)	−0.001 (3)	0.011 (4)
C50	0.044 (4)	0.096 (6)	0.034 (4)	−0.022 (4)	0.008 (3)	0.004 (4)
C51	0.052 (4)	0.067 (4)	0.031 (3)	−0.015 (3)	−0.001 (3)	0.007 (3)
C52	0.036 (3)	0.048 (3)	0.036 (3)	−0.010 (3)	0.001 (3)	0.003 (3)
C53	0.026 (3)	0.032 (3)	0.022 (3)	−0.001 (2)	−0.006 (2)	−0.001 (2)
C54	0.052 (4)	0.035 (3)	0.029 (3)	0.001 (3)	−0.004 (3)	−0.007 (2)
S1	0.0557 (15)	0.0781 (17)	0.0728 (18)	0.0190 (13)	−0.0043 (12)	−0.0238 (14)
F1	0.45 (2)	0.272 (15)	0.179 (11)	0.273 (17)	−0.189 (14)	−0.138 (11)
F2	0.49 (3)	0.081 (7)	0.270 (17)	0.020 (11)	−0.152 (18)	−0.030 (9)
F3	0.090 (7)	0.363 (19)	0.223 (14)	0.031 (9)	−0.042 (8)	−0.043 (14)
O3	0.048 (3)	0.040 (3)	0.064 (4)	0.003 (3)	0.010 (3)	−0.023 (3)
O4	0.067 (4)	0.116 (6)	0.059 (4)	0.057 (4)	−0.026 (4)	−0.017 (4)
O5	0.230 (10)	0.052 (4)	0.027 (3)	0.059 (5)	0.022 (5)	−0.010 (3)
C55	0.0557 (15)	0.0781 (17)	0.0728 (18)	0.0190 (13)	−0.0043 (12)	−0.0238 (14)
Cl1	0.063 (6)	0.133 (9)	0.127 (9)	−0.010 (6)	0.005 (6)	0.036 (8)

### Geometric parameters (Å, º)

**Table:**

Ru1—O1	2.191 (3)	C25—C26	1.383 (8)
Ru1—O2	2.202 (3)	C25—H25	0.9500
Ru1—P3	2.2881 (13)	C26—H26	0.9500
Ru1—P1	2.3211 (13)	C27—C28	1.386 (7)
Ru1—P2	2.3680 (13)	C27—C32	1.397 (7)
Ru1—P4	2.3791 (13)	C28—C29	1.398 (7)
P1—C13	1.841 (5)	C28—H28	0.9500
P1—C1	1.841 (5)	C29—C30	1.368 (8)
P1—C7	1.844 (5)	C29—H29	0.9500
P2—C21	1.827 (5)	C30—C31	1.372 (8)
P2—C14	1.837 (5)	C30—H30	0.9500
P2—C15	1.837 (5)	C31—C32	1.385 (8)
P3—C33	1.828 (5)	C31—H31	0.9500
P3—C27	1.841 (5)	C32—H32	0.9500
P3—C39	1.846 (5)	C33—C38	1.390 (7)
P4—C47	1.825 (5)	C33—C34	1.394 (7)
P4—C40	1.840 (5)	C34—C35	1.389 (7)
P4—C41	1.846 (5)	C34—H34	0.9500
O1—C53	1.267 (6)	C35—C36	1.384 (8)
O2—C53	1.262 (6)	C35—H35	0.9500
C1—C2	1.386 (8)	C36—C37	1.379 (8)
C1—C6	1.391 (8)	C36—H36	0.9500
C2—C3	1.394 (8)	C37—C38	1.382 (7)
C2—H2	0.9500	C37—H37	0.9500
C3—C4	1.378 (9)	C38—H38	0.9500
C3—H3	0.9500	C39—C40	1.521 (7)
C4—C5	1.379 (10)	C39—H39A	0.9900
C4—H4	0.9500	C39—H39B	0.9900
C5—C6	1.388 (8)	C40—H40A	0.9900
C5—H5	0.9500	C40—H40B	0.9900
C6—H6	0.9500	C41—C46	1.397 (8)
C7—C12	1.389 (7)	C41—C42	1.405 (8)
C7—C8	1.398 (7)	C42—C43	1.392 (8)
C8—C9	1.383 (7)	C42—H42	0.9500
C8—H8	0.9500	C43—C44	1.380 (9)
C9—C10	1.387 (8)	C43—H43	0.9500
C9—H9	0.9500	C44—C45	1.381 (10)
C10—C11	1.382 (7)	C44—H44	0.9500
C10—H10	0.9500	C45—C46	1.380 (9)
C11—C12	1.371 (7)	C45—H45	0.9500
C11—H11	0.9500	C46—H46	0.9500
C12—H12	0.9500	C47—C52	1.389 (8)
C13—C14	1.530 (7)	C47—C48	1.395 (8)
C13—H13A	0.9900	C48—C49	1.384 (9)
C13—H13B	0.9900	C48—H48	0.9500
C14—H14A	0.9900	C49—C50	1.366 (10)
C14—H14B	0.9900	C49—H49	0.9500
C15—C16	1.381 (8)	C50—C51	1.381 (10)
C15—C20	1.397 (8)	C50—H50	0.9500
C16—C17	1.386 (9)	C51—C52	1.402 (8)
C16—H16	0.9500	C51—H51	0.9500
C17—C18	1.379 (10)	C52—H52	0.9500
C17—H17	0.9500	C53—C54	1.505 (7)
C18—C19	1.381 (9)	C54—H54A	0.9800
C18—H18	0.9500	C54—H54B	0.9800
C19—C20	1.388 (8)	C54—H54C	0.9800
C19—H19	0.9500	C54—H54D	0.9800
C20—H20	0.9500	C54—H54E	0.9800
C21—C22	1.376 (8)	C54—H54F	0.9800
C21—C26	1.400 (7)	S1—O4	1.343 (6)
C22—C23	1.390 (8)	S1—O5	1.394 (6)
C22—H22	0.9500	S1—O3	1.434 (6)
C23—C24	1.377 (9)	S1—C55	1.8204 (10)
C23—H23	0.9500	F1—C55	1.3206 (10)
C24—C25	1.372 (9)	F2—C55	1.3209 (10)
C24—H24	0.9500	F3—C55	1.3207 (10)
			
O1—Ru1—O2	59.18 (12)	C26—C25—H25	119.8
O1—Ru1—P3	105.48 (10)	C25—C26—C21	120.4 (5)
O2—Ru1—P3	160.60 (9)	C25—C26—H26	119.8
O1—Ru1—P1	161.81 (10)	C21—C26—H26	119.8
O2—Ru1—P1	106.97 (9)	C28—C27—C32	118.8 (5)
P3—Ru1—P1	90.34 (5)	C28—C27—P3	122.9 (4)
O1—Ru1—P2	85.62 (10)	C32—C27—P3	118.1 (4)
O2—Ru1—P2	89.53 (9)	C27—C28—C29	120.3 (5)
P3—Ru1—P2	101.66 (5)	C27—C28—H28	119.8
P1—Ru1—P2	82.36 (5)	C29—C28—H28	119.8
O1—Ru1—P4	88.08 (10)	C30—C29—C28	119.9 (5)
O2—Ru1—P4	84.19 (9)	C30—C29—H29	120.1
P3—Ru1—P4	83.44 (5)	C28—C29—H29	120.1
P1—Ru1—P4	102.89 (5)	C29—C30—C31	120.5 (5)
P2—Ru1—P4	172.77 (5)	C29—C30—H30	119.7
O1—Ru1—C53	29.65 (14)	C31—C30—H30	119.7
O2—Ru1—C53	29.55 (14)	C30—C31—C32	120.2 (6)
P3—Ru1—C53	133.90 (12)	C30—C31—H31	119.9
P1—Ru1—C53	135.75 (12)	C32—C31—H31	119.9
P2—Ru1—C53	87.98 (11)	C31—C32—C27	120.3 (5)
P4—Ru1—C53	84.79 (11)	C31—C32—H32	119.9
C13—P1—C1	102.4 (3)	C27—C32—H32	119.9
C13—P1—C7	99.9 (2)	C38—C33—C34	118.7 (5)
C1—P1—C7	99.7 (2)	C38—C33—P3	122.5 (4)
C13—P1—Ru1	107.82 (16)	C34—C33—P3	118.8 (4)
C1—P1—Ru1	127.97 (18)	C35—C34—C33	121.0 (5)
C7—P1—Ru1	115.12 (17)	C35—C34—H34	119.5
C21—P2—C14	106.9 (2)	C33—C34—H34	119.5
C21—P2—C15	102.9 (2)	C36—C35—C34	119.4 (5)
C14—P2—C15	105.0 (3)	C36—C35—H35	120.3
C21—P2—Ru1	119.04 (17)	C34—C35—H35	120.3
C14—P2—Ru1	109.69 (18)	C37—C36—C35	119.9 (5)
C15—P2—Ru1	112.26 (17)	C37—C36—H36	120.1
C33—P3—C27	100.5 (2)	C35—C36—H36	120.1
C33—P3—C39	104.8 (2)	C36—C37—C38	120.8 (5)
C27—P3—C39	98.2 (2)	C36—C37—H37	119.6
C33—P3—Ru1	124.40 (16)	C38—C37—H37	119.6
C27—P3—Ru1	118.19 (17)	C37—C38—C33	120.2 (5)
C39—P3—Ru1	107.13 (17)	C37—C38—H38	119.9
C47—P4—C40	108.1 (3)	C33—C38—H38	119.9
C47—P4—C41	100.8 (2)	C40—C39—P3	108.2 (3)
C40—P4—C41	104.9 (2)	C40—C39—H39A	110.1
C47—P4—Ru1	118.71 (18)	P3—C39—H39A	110.1
C40—P4—Ru1	107.88 (17)	C40—C39—H39B	110.1
C41—P4—Ru1	115.37 (17)	P3—C39—H39B	110.1
C53—O1—Ru1	91.5 (3)	H39A—C39—H39B	108.4
C53—O2—Ru1	91.1 (3)	C39—C40—P4	109.7 (3)
C2—C1—C6	118.2 (5)	C39—C40—H40A	109.7
C2—C1—P1	119.2 (4)	P4—C40—H40A	109.7
C6—C1—P1	122.5 (5)	C39—C40—H40B	109.7
C1—C2—C3	121.4 (6)	P4—C40—H40B	109.7
C1—C2—H2	119.3	H40A—C40—H40B	108.2
C3—C2—H2	119.3	C46—C41—C42	118.4 (5)
C4—C3—C2	119.8 (6)	C46—C41—P4	121.7 (4)
C4—C3—H3	120.1	C42—C41—P4	119.7 (4)
C2—C3—H3	120.1	C43—C42—C41	120.3 (6)
C3—C4—C5	119.4 (6)	C43—C42—H42	119.8
C3—C4—H4	120.3	C41—C42—H42	119.8
C5—C4—H4	120.3	C44—C43—C42	120.3 (6)
C4—C5—C6	120.9 (6)	C44—C43—H43	119.8
C4—C5—H5	119.5	C42—C43—H43	119.8
C6—C5—H5	119.5	C43—C44—C45	119.6 (6)
C5—C6—C1	120.3 (6)	C43—C44—H44	120.2
C5—C6—H6	119.8	C45—C44—H44	120.2
C1—C6—H6	119.8	C46—C45—C44	121.0 (6)
C12—C7—C8	117.8 (5)	C46—C45—H45	119.5
C12—C7—P1	122.3 (4)	C44—C45—H45	119.5
C8—C7—P1	119.8 (4)	C45—C46—C41	120.4 (6)
C9—C8—C7	120.8 (5)	C45—C46—H46	119.8
C9—C8—H8	119.6	C41—C46—H46	119.8
C7—C8—H8	119.6	C52—C47—C48	119.7 (5)
C8—C9—C10	120.4 (5)	C52—C47—P4	117.3 (4)
C8—C9—H9	119.8	C48—C47—P4	123.0 (4)
C10—C9—H9	119.8	C49—C48—C47	119.4 (6)
C11—C10—C9	119.0 (5)	C49—C48—H48	120.3
C11—C10—H10	120.5	C47—C48—H48	120.3
C9—C10—H10	120.5	C50—C49—C48	121.3 (7)
C12—C11—C10	120.7 (5)	C50—C49—H49	119.3
C12—C11—H11	119.7	C48—C49—H49	119.3
C10—C11—H11	119.7	C49—C50—C51	120.0 (6)
C11—C12—C7	121.4 (5)	C49—C50—H50	120.0
C11—C12—H12	119.3	C51—C50—H50	120.0
C7—C12—H12	119.3	C50—C51—C52	119.8 (6)
C14—C13—P1	108.3 (3)	C50—C51—H51	120.1
C14—C13—H13A	110.0	C52—C51—H51	120.1
P1—C13—H13A	110.0	C47—C52—C51	119.7 (6)
C14—C13—H13B	110.0	C47—C52—H52	120.1
P1—C13—H13B	110.0	C51—C52—H52	120.1
H13A—C13—H13B	108.4	O2—C53—O1	118.1 (4)
C13—C14—P2	109.5 (3)	O2—C53—C54	120.9 (5)
C13—C14—H14A	109.8	O1—C53—C54	121.0 (5)
P2—C14—H14A	109.8	O2—C53—Ru1	59.4 (2)
C13—C14—H14B	109.8	O1—C53—Ru1	58.8 (2)
P2—C14—H14B	109.8	C54—C53—Ru1	177.0 (4)
H14A—C14—H14B	108.2	C53—C54—H54A	109.5
C16—C15—C20	118.6 (5)	C53—C54—H54B	109.5
C16—C15—P2	121.0 (4)	H54A—C54—H54B	109.5
C20—C15—P2	120.3 (4)	C53—C54—H54C	109.5
C15—C16—C17	120.3 (6)	H54A—C54—H54C	109.5
C15—C16—H16	119.8	H54B—C54—H54C	109.5
C17—C16—H16	119.8	C53—C54—H54D	109.5
C18—C17—C16	121.1 (6)	H54A—C54—H54D	141.1
C18—C17—H17	119.4	H54B—C54—H54D	56.3
C16—C17—H17	119.4	H54C—C54—H54D	56.3
C17—C18—C19	118.9 (6)	C53—C54—H54E	109.5
C17—C18—H18	120.5	H54A—C54—H54E	56.3
C19—C18—H18	120.5	H54B—C54—H54E	141.1
C18—C19—C20	120.4 (6)	H54C—C54—H54E	56.3
C18—C19—H19	119.8	H54D—C54—H54E	109.5
C20—C19—H19	119.8	C53—C54—H54F	109.5
C19—C20—C15	120.6 (6)	H54A—C54—H54F	56.3
C19—C20—H20	119.7	H54B—C54—H54F	56.3
C15—C20—H20	119.7	H54C—C54—H54F	141.1
C22—C21—C26	118.5 (5)	H54D—C54—H54F	109.5
C22—C21—P2	123.7 (4)	H54E—C54—H54F	109.5
C26—C21—P2	117.8 (4)	O4—S1—O5	108.7 (5)
C21—C22—C23	120.8 (6)	O4—S1—O3	118.6 (5)
C21—C22—H22	119.6	O5—S1—O3	107.0 (4)
C23—C22—H22	119.6	O4—S1—C55	96.1 (5)
C24—C23—C22	120.0 (6)	O5—S1—C55	117.8 (5)
C24—C23—H23	120.0	O3—S1—C55	108.9 (3)
C22—C23—H23	120.0	F1—C55—F3	108.95 (12)
C25—C24—C23	119.9 (6)	F1—C55—F2	108.94 (12)
C25—C24—H24	120.0	F3—C55—F2	108.90 (12)
C23—C24—H24	120.0	F1—C55—S1	105.2 (8)
C24—C25—C26	120.3 (6)	F3—C55—S1	114.9 (7)
C24—C25—H25	119.8	F2—C55—S1	109.8 (8)
			
O1—Ru1—P1—C13	71.7 (4)	Ru1—P2—C15—C16	−86.8 (5)
O2—Ru1—P1—C13	109.8 (2)	C21—P2—C15—C20	−39.5 (5)
P3—Ru1—P1—C13	−79.07 (18)	C14—P2—C15—C20	−151.2 (4)
P2—Ru1—P1—C13	22.64 (18)	Ru1—P2—C15—C20	89.7 (4)
P4—Ru1—P1—C13	−162.41 (18)	C20—C15—C16—C17	−0.8 (9)
C53—Ru1—P1—C13	101.8 (2)	P2—C15—C16—C17	175.7 (5)
O1—Ru1—P1—C1	−165.8 (4)	C15—C16—C17—C18	−0.6 (10)
O2—Ru1—P1—C1	−127.7 (2)	C16—C17—C18—C19	1.3 (10)
P3—Ru1—P1—C1	43.4 (2)	C17—C18—C19—C20	−0.6 (9)
P2—Ru1—P1—C1	145.1 (2)	C18—C19—C20—C15	−0.8 (9)
P4—Ru1—P1—C1	−40.0 (2)	C16—C15—C20—C19	1.4 (8)
C53—Ru1—P1—C1	−135.8 (3)	P2—C15—C20—C19	−175.1 (5)
O1—Ru1—P1—C7	−38.7 (4)	C14—P2—C21—C22	14.6 (5)
O2—Ru1—P1—C7	−0.7 (2)	C15—P2—C21—C22	−95.7 (5)
P3—Ru1—P1—C7	170.47 (17)	Ru1—P2—C21—C22	139.5 (4)
P2—Ru1—P1—C7	−87.81 (17)	C14—P2—C21—C26	−164.1 (4)
P4—Ru1—P1—C7	87.13 (18)	C15—P2—C21—C26	85.6 (4)
C53—Ru1—P1—C7	−8.7 (2)	Ru1—P2—C21—C26	−39.3 (4)
O1—Ru1—P2—C21	71.3 (2)	C26—C21—C22—C23	−1.6 (9)
O2—Ru1—P2—C21	130.4 (2)	P2—C21—C22—C23	179.7 (5)
P3—Ru1—P2—C21	−33.60 (19)	C21—C22—C23—C24	1.8 (11)
P1—Ru1—P2—C21	−122.36 (19)	C22—C23—C24—C25	−0.9 (11)
C53—Ru1—P2—C21	100.9 (2)	C23—C24—C25—C26	−0.1 (10)
O1—Ru1—P2—C14	−165.2 (2)	C24—C25—C26—C21	0.3 (8)
O2—Ru1—P2—C14	−106.1 (2)	C22—C21—C26—C25	0.6 (8)
P3—Ru1—P2—C14	89.9 (2)	P2—C21—C26—C25	179.3 (4)
P1—Ru1—P2—C14	1.1 (2)	C33—P3—C27—C28	143.8 (4)
C53—Ru1—P2—C14	−135.6 (2)	C39—P3—C27—C28	−109.4 (5)
O1—Ru1—P2—C15	−48.9 (2)	Ru1—P3—C27—C28	5.1 (5)
O2—Ru1—P2—C15	10.2 (2)	C33—P3—C27—C32	−42.2 (5)
P3—Ru1—P2—C15	−153.83 (19)	C39—P3—C27—C32	64.5 (5)
P1—Ru1—P2—C15	117.4 (2)	Ru1—P3—C27—C32	179.0 (4)
C53—Ru1—P2—C15	−19.3 (2)	C32—C27—C28—C29	0.2 (8)
O1—Ru1—P3—C33	−128.3 (2)	P3—C27—C28—C29	174.1 (4)
O2—Ru1—P3—C33	−163.8 (3)	C27—C28—C29—C30	−0.9 (9)
P1—Ru1—P3—C33	42.56 (19)	C28—C29—C30—C31	1.5 (10)
P2—Ru1—P3—C33	−39.7 (2)	C29—C30—C31—C32	−1.3 (12)
P4—Ru1—P3—C33	145.49 (19)	C30—C31—C32—C27	0.5 (11)
C53—Ru1—P3—C33	−138.3 (2)	C28—C27—C32—C31	0.0 (9)
O1—Ru1—P3—C27	−0.1 (2)	P3—C27—C32—C31	−174.2 (5)
O2—Ru1—P3—C27	−35.6 (4)	C27—P3—C33—C38	127.6 (4)
P1—Ru1—P3—C27	170.76 (19)	C39—P3—C33—C38	26.1 (5)
P2—Ru1—P3—C27	88.49 (19)	Ru1—P3—C33—C38	−97.2 (4)
P4—Ru1—P3—C27	−86.31 (19)	C27—P3—C33—C34	−53.4 (4)
C53—Ru1—P3—C27	−10.1 (3)	C39—P3—C33—C34	−154.9 (4)
O1—Ru1—P3—C39	109.4 (2)	Ru1—P3—C33—C34	81.8 (4)
O2—Ru1—P3—C39	73.9 (3)	C38—C33—C34—C35	2.0 (8)
P1—Ru1—P3—C39	−79.72 (18)	P3—C33—C34—C35	−177.1 (4)
P2—Ru1—P3—C39	−161.99 (18)	C33—C34—C35—C36	−1.0 (9)
P4—Ru1—P3—C39	23.22 (18)	C34—C35—C36—C37	−0.1 (9)
C53—Ru1—P3—C39	99.5 (2)	C35—C36—C37—C38	0.2 (9)
O1—Ru1—P4—C47	131.8 (2)	C36—C37—C38—C33	0.8 (8)
O2—Ru1—P4—C47	72.6 (2)	C34—C33—C38—C37	−1.8 (8)
P3—Ru1—P4—C47	−122.4 (2)	P3—C33—C38—C37	177.2 (4)
P1—Ru1—P4—C47	−33.6 (2)	C33—P3—C39—C40	176.6 (3)
C53—Ru1—P4—C47	102.2 (2)	C27—P3—C39—C40	73.3 (4)
O1—Ru1—P4—C40	−104.9 (2)	Ru1—P3—C39—C40	−49.6 (4)
O2—Ru1—P4—C40	−164.1 (2)	P3—C39—C40—P4	51.2 (4)
P3—Ru1—P4—C40	0.93 (18)	C47—P4—C40—C39	98.5 (4)
P1—Ru1—P4—C40	89.78 (19)	C41—P4—C40—C39	−154.5 (4)
C53—Ru1—P4—C40	−134.4 (2)	Ru1—P4—C40—C39	−31.0 (4)
O1—Ru1—P4—C41	12.0 (2)	C47—P4—C41—C46	−18.4 (5)
O2—Ru1—P4—C41	−47.2 (2)	C40—P4—C41—C46	−130.7 (5)
P3—Ru1—P4—C41	117.8 (2)	Ru1—P4—C41—C46	110.8 (5)
P1—Ru1—P4—C41	−153.4 (2)	C47—P4—C41—C42	165.6 (4)
C53—Ru1—P4—C41	−17.5 (2)	C40—P4—C41—C42	53.3 (5)
O2—Ru1—O1—C53	1.5 (3)	Ru1—P4—C41—C42	−65.2 (4)
P3—Ru1—O1—C53	−165.5 (3)	C46—C41—C42—C43	1.0 (8)
P1—Ru1—O1—C53	44.9 (5)	P4—C41—C42—C43	177.2 (4)
P2—Ru1—O1—C53	93.6 (3)	C41—C42—C43—C44	−0.7 (8)
P4—Ru1—O1—C53	−82.8 (3)	C42—C43—C44—C45	0.3 (9)
O1—Ru1—O2—C53	−1.6 (3)	C43—C44—C45—C46	−0.2 (10)
P3—Ru1—O2—C53	39.1 (4)	C44—C45—C46—C41	0.5 (10)
P1—Ru1—O2—C53	−168.6 (3)	C42—C41—C46—C45	−0.9 (9)
P2—Ru1—O2—C53	−86.7 (3)	P4—C41—C46—C45	−177.0 (5)
P4—Ru1—O2—C53	89.7 (3)	C40—P4—C47—C52	−160.4 (4)
C13—P1—C1—C2	178.6 (4)	C41—P4—C47—C52	89.8 (5)
C7—P1—C1—C2	−79.0 (4)	Ru1—P4—C47—C52	−37.2 (5)
Ru1—P1—C1—C2	53.9 (5)	C40—P4—C47—C48	20.1 (6)
C13—P1—C1—C6	−5.5 (5)	C41—P4—C47—C48	−89.6 (5)
C7—P1—C1—C6	97.0 (4)	Ru1—P4—C47—C48	143.3 (5)
Ru1—P1—C1—C6	−130.1 (4)	C52—C47—C48—C49	2.1 (10)
C6—C1—C2—C3	1.1 (8)	P4—C47—C48—C49	−178.4 (5)
P1—C1—C2—C3	177.2 (4)	C47—C48—C49—C50	−1.4 (11)
C1—C2—C3—C4	−1.9 (9)	C48—C49—C50—C51	−0.2 (12)
C2—C3—C4—C5	1.0 (9)	C49—C50—C51—C52	1.0 (11)
C3—C4—C5—C6	0.6 (9)	C48—C47—C52—C51	−1.3 (9)
C4—C5—C6—C1	−1.4 (9)	P4—C47—C52—C51	179.2 (5)
C2—C1—C6—C5	0.6 (8)	C50—C51—C52—C47	−0.2 (9)
P1—C1—C6—C5	−175.4 (4)	Ru1—O2—C53—O1	2.6 (4)
C13—P1—C7—C12	−105.0 (4)	Ru1—O2—C53—C54	−176.5 (4)
C1—P1—C7—C12	150.5 (4)	Ru1—O1—C53—O2	−2.6 (4)
Ru1—P1—C7—C12	10.1 (5)	Ru1—O1—C53—C54	176.5 (4)
C13—P1—C7—C8	72.6 (4)	O1—Ru1—C53—O2	177.3 (5)
C1—P1—C7—C8	−31.9 (5)	P3—Ru1—C53—O2	−163.1 (2)
Ru1—P1—C7—C8	−172.3 (3)	P1—Ru1—C53—O2	15.7 (3)
C12—C7—C8—C9	−1.3 (8)	P2—Ru1—C53—O2	92.6 (3)
P1—C7—C8—C9	−179.0 (4)	P4—Ru1—C53—O2	−87.4 (3)
C7—C8—C9—C10	0.4 (8)	O2—Ru1—C53—O1	−177.3 (5)
C8—C9—C10—C11	1.5 (8)	P3—Ru1—C53—O1	19.6 (3)
C9—C10—C11—C12	−2.5 (8)	P1—Ru1—C53—O1	−161.6 (2)
C10—C11—C12—C7	1.6 (8)	P2—Ru1—C53—O1	−84.7 (3)
C8—C7—C12—C11	0.3 (8)	P4—Ru1—C53—O1	95.3 (3)
P1—C7—C12—C11	177.9 (4)	O4—S1—C55—F1	−64.2 (5)
C1—P1—C13—C14	174.7 (3)	O5—S1—C55—F1	−179.1 (5)
C7—P1—C13—C14	72.3 (4)	O3—S1—C55—F1	58.9 (5)
Ru1—P1—C13—C14	−48.2 (4)	O4—S1—C55—F3	176.0 (6)
P1—C13—C14—P2	49.5 (4)	O5—S1—C55—F3	61.1 (6)
C21—P2—C14—C13	100.1 (4)	O3—S1—C55—F3	−60.9 (6)
C15—P2—C14—C13	−151.1 (4)	O4—S1—C55—F2	52.9 (6)
Ru1—P2—C14—C13	−30.3 (4)	O5—S1—C55—F2	−62.1 (6)
C21—P2—C15—C16	144.0 (5)	O3—S1—C55—F2	176.0 (5)
C14—P2—C15—C16	32.3 (5)		
